# Prospective, multicenter study of P4HB (Phasix™) mesh for hernia repair in cohort at risk for complications: 3-Year follow-up

**DOI:** 10.1016/j.amsu.2020.12.002

**Published:** 2020-12-15

**Authors:** John Scott Roth, Gary J. Anthone, Don J. Selzer, Benjamin K. Poulose, Richard A. Pierce, James G. Bittner, William W. Hope, Raymond M. Dunn, Robert G. Martindale, Matthew I. Goldblatt, David B. Earle, John R. Romanelli, Gregory J. Mancini, Jacob A. Greenberg, John G. Linn, Eduardo Parra-Davila, Bryan J. Sandler, Corey R. Deeken, Jasenka Verbarg, Jennifer L. Salluzzo, Guy R. Voeller

**Affiliations:** a Department of Surgery, University of Kentucky Medical Center, Lexington, KY, USA; b Department of Surgery, Methodist Health System, Omaha, NE, USA; c Department of Surgery, Indiana University School of Medicine, Indianapolis, IN, USA; d Center for Abdominal Core Health, The Ohio State Wexner Medical Center, Columbus, OH, USA; e Department of Surgery, Vanderbilt University Medical Center, Nashville, TN, USA; f Department of Surgery, Saint Francis Hospital, Hartford, CT, USA; g Department of Surgery, New Hanover Regional Medical Center, Wilmington, NC, USA; h Department of Surgery, University of Massachusetts Worcester, Worcester, MA, USA; i Department of Surgery, Oregon Health & Science University, Portland, OR, USA; j Department of Surgery, The Medical College of Wisconsin, Inc., Milwaukee, WI, USA; k Department of Surgery, New England Hernia Center, Lowell, MA, USA; l Department of Surgery, Baystate Medical Center, Springfield, MA, USA; m Department of Surgery, The University of Tennessee Health Science Center, Memphis, TN, USA; n Department of Surgery, Board of Regents of the University of Wisconsin System, Madison, WI, USA; o Department of Surgery, North Shore University Health System, Evanston, IL, USA; p Department of Surgery, Celebration Health, Celebration, FL, USA; q Department of Surgery, The Regents of the University of California, San Diego, CA, USA; r Covalent Bio, LLC, St. Louis, MO, USA; s Department of Surgery, Virginia Commonwealth University, Richmond, VA, USA

**Keywords:** Hernia repair, Recurrence, Infection, Poly-4-hydroxybutyrate, Mesh, Seroma

## Abstract

**Background:**

This study represents a prospective, multicenter, open-label study to assess the safety, performance, and outcomes of poly-4-hydroxybutyrate (P4HB, Phasix™) mesh for primary ventral, primary incisional, or multiply-recurrent hernia in subjects at risk for complications. This study reports 3-year clinical outcomes.

**Materials and methods:**

P4HB mesh was implanted in 121 patients via retrorectus or onlay technique. Physical exam and/or quality of life surveys were completed at 1, 3, 6,12, 18, 24, and 36 months, with 5-year (60-month) follow-up ongoing.

**Results:**

A total of n = 121 patients were implanted with P4HB mesh (n = 75 (62%) female) with a mean age of 54.7 ± 12.0 years and mean BMI of 32.2 ± 4.5 kg/m^2^ (±standard deviation). Comorbidities included: obesity (78.5%), active smokers (23.1%), COPD (28.1%), diabetes mellitus (33.1%), immunosuppression (8.3%), coronary artery disease (21.5%), chronic corticosteroid use (5.0%), hypo-albuminemia (2.5%), advanced age (5.0%), and renal insufficiency (0.8%). Hernias were repaired via retrorectus (n = 45, 37.2% with myofascial release (MR) or n = 43, 35.5% without MR), onlay (n = 8, 6.6% with MR or n = 24, 19.8% without MR), or not reported (n = 1, 0.8%). 82 patients (67.8%) completed 36-month follow-up. 17 patients (17.9% ± 0.4%) experienced hernia recurrence at 3 years, with n = 9 in the retrorectus group and n = 8 in the onlay group. SSI (n = 11) occurred in 9.3% ± 0.03% of patients.

**Conclusions:**

Long-term outcomes following ventral hernia repair with P4HB mesh demonstrate low recurrence rates at 3-year (36-month) postoperative time frame with no patients developing late mesh complications or requiring mesh removal. 5-year (60-month) follow-up is ongoing.

## Highlights

• Prospective, multicenter, open-label study of safety, performance, and outcomes.
• Poly-4-hydroxybutyrate mesh implanted in n = 121 patients via retrorectus or onlay.
• 82 patients (67.8%) completed 36-month follow-up.
• Hernia recurrence (n = 17, 17.9% ± 0.4%) and SSI (n = 11, 9.3% ± 0.03%) at 3 years.
• No patients developed late mesh complications or required mesh removal.

## 1 Introduction

Ventral hernia repair remains one of the most common and challenging general surgical procedures due to variations in surgical technique and patient characteristics/comorbidities. The selection of a biomaterial to repair the abdominal wall contributes to the complexity of the repair, with over 150 devices marketed for this application [1]. Historically, permanent synthetic materials were utilized, followed by biological tissue-derived materials, and most recently, absorbable materials such as polyglycolide, polylactide, trimethylene carbonate, and poly-4-hydroxybutyrate (P4HB) [[2], [3], [4]].

P4HB has received regulatory clearance for use in sutures, as well as in medical devices for hernia repair, orthopedic applications, and plastic/reconstructive surgery [5]. P4HB mesh has been used in several retrospective and prospective clinical studies for hernia repair with medium (18–24 months) to long-term (36+ months) outcomes evaluated [[6], [7], [8], [9], [10]]. P4HB offers a long-term resorption profile of 12–18 months, providing mechanical support of the defect to prevent early hernia recurrence [11,12]. It is unknown how frequently this particular device is utilized compared to other similar devices, but some surgeons estimate that resorbable hernia repair materials are currently utilized in approximately 5% of hernia repair cases (*personal communication*). Resorbable materials such as P4HB have also been utilized for incisional hernia prophylaxis [13].

P4HB mesh has also been characterized in several preclinical [11,12,14] studies. In a 52-week porcine study, P4HB repairs demonstrated a consistent strength profile, with mesh-repair strengths significantly greater than the native abdominal wall over time, despite significant resorption of the P4HB [11]. In a porcine study by Martin et al., P4HB fibers exhibited significant decrease in fiber diameter and molecular weight, indicating bulk degradation of the polymer throughout the 72 week study [12]. P4HB fibers displayed evidence of degradation at 48 weeks, and only small fragments were visible at 72 weeks. However, mechanical testing revealed similar strengths between P4HB mesh-repaired sites and native porcine abdominal wall at 72 weeks. The data from these preclinical studies suggest that P4HB mesh contributes to the strength of the porcine abdominal wall for approximately 1 year after implantation and contributes negligible strength at 18 months.

## 2 Methods

### 2.1 Study design

This study represents a prospective, multicenter, open-label study to assess safety, performance, and outcomes of P4HB mesh (Phasix™ Mesh, C.R. Bard, Inc., Warwick, RI) for primary ventral or incisional or multiply-recurrent hernia repair in a cohort at risk for complications. This study has been registered with ClinicalTrials.gov (ClinicalTrials.gov/NCT01961687). The primary aim of this study is to evaluate 36-month outcomes among patients undergoing hernia repair with P4HB mesh. The 36-month outcomes reported here are well beyond the 18–24 month timeframe reported in the peer-reviewed literature for similar resorbable mesh products [[15], [16], [17]], making this study particularly unique and relevant to hernia surgeons. This study provides important insight into the long-term performance of P4HB mesh at a time point in which the mesh itself is no longer contributing to the mechanical strength of the repair. At 36 months postimplantation, all of the repair strength is dependent upon the strength of the native abdominal wall in combination with the host tissue that has been regenerated at the repair site.

Methods were previously described and are repeated here for clarity [8]. Subjects were considered at risk for complications with one or more of the following comorbidities: body mass index (BMI) between 30 and 40 kg/m^2^ (inclusive), active smokers, chronic obstructive pulmonary disease (COPD), diabetes mellitus, immunosuppression, coronary artery disease, chronic corticosteroid use (>6 months systemic use), hypo-albuminemia (pre-operative serum albumin <3.4 g/dL), advanced age (≥75 years), or renal insufficiency (serum creatinine concentration ≥ 2.5 mg/dL). Subjects, investigators, and surgeons were not blinded to study treatment. The study was designed to treat 120 subjects at 16 U S. sites. The protocol was approved by the Institutional Review Board (IRB) at each institution, and all subjects provided informed consent prior to enrollment. Recruitment occurred through the surgical offices of the Investigators based on the eligibility criteria between October 2013 and January 2015.

### 2.2 Inclusion/exclusion criteria

Subjects ≥18 years of age, with primary ventral, primary incisional, or recurrent incisional hernia (not to exceed 3 recurrences) were evaluated for eligibility, including: one or more comorbidities listed above, Class I surgical wound (defined by Centers for Disease Control and Prevention (CDC)) [18], and 10–350 cm^2^ hernia defect suitable for repair via retrorectus or onlay mesh (with or without myofascial release, MR). Exclusion criteria included: four or more previous hernia repairs (of the index repair), peritonitis, on or anticipated to be placed on chemotherapy during study period, BMI > 40 kg/m^2^, cirrhosis of the liver and/or ascites, American Society of Anesthesiology Class 4 or 5, diagnosed human immunodeficiency virus (HIV) infection, life expectancy of less than 2 years at time of enrollment, planned intra-abdominal mesh placement or bridged repair, surgical wound designated Class II (clean-contaminated), Class III (contaminated) or Class IV (dirty-contaminated) defined by CDC [18] (no device is currently indicated for use in contaminated or infected fields), active or latent systemic infection, pregnant or plans to become pregnant during study period, currently breastfeeding, enrolled in another clinical study within last 30 days, part of site personnel directly involved with study, known allergy to test device or component materials, or any condition that, in the opinion of the Investigator, would preclude the use of the study device, or preclude the subject from completing the follow-up requirements.

### 2.3 Surgical technique

All subjects were administered antibiotics according to hospital protocol and underwent open ventral hernia repair. Intraoperative inclusion and exclusion criteria were assessed and documented. Subjects meeting intraoperative eligibility criteria received P4HB mesh, overlapping the defect by at least 5 cm with 6–12 resorbable sutures at approximately 5–6 cm intervals around the periphery. The hernia defect was closed by approximating the fascial edges, including additional myofascial release, if required. The fascial and subcutaneous layers were closed with sutures, and the skin was closed with staples and/or sutures. Operative details including hernia defect size, mesh size, mesh position, repair technique, use of myofascial release, suture type, number of sutures to secure mesh, and procedural time were collected. Investigators were selected based upon experience with hernia repair techniques. No specific training was required for participation due to the similarity in technique required for P4HB mesh relative to other meshes. Postoperative care was performed consistent with surgeon practice at each site.

### 2.4 Data Collection

Postoperative patient visits were scheduled at 1, 3, 6,12, 18, 24, 36, and 60 months, and a telephone interview was conducted at 30 months. Medical history, demographic information, and all current prescription and over the counter (OTC) pain medications were recorded. The Pain Visual Analogue Scale (VAS) and quality of life assessments: Carolinas Comfort Scale® (CCS) and 12-Item Short Form Health Survey® (SF-12) were completed preoperatively and at scheduled intervals, along with physical examination to assess hernia recurrence, surgical complications, and adverse events.

### 2.5 Study endpoints

Primary endpoints included: hernia recurrence and surgical site infections (SSI). Hernia recurrence was assessed by physical examination at each study visit. A recurrent hernia was defined as any hernia identified or confirmed by the investigator, during any study follow-up visit, within 7 cm of the repair. Hernia recurrence identified via incidental magnetic resonance imaging (MRI) or computed tomography (CT) scan were evaluated by the operating surgeon for clinical significance and confirmation.

SSI was assessed by physical examination with confirmation by gram stain and culture. Superficial and deep SSI were classified according to CDC guidelines [19]. SSI may occur due to contamination during the surgical procedure, factors related to surgical technique (i.e. tissue plane, component separation, inadvertent enterotomy, etc.) or even patient comorbidities (i.e. obesity, smoking, diabetes, etc.) [20]. The CDC criteria indicate that SSIs are typically diagnosed within the first 30–90 days [18]. However, the peer-reviewed literature has documented cases of SSI several years after implantation [20,21]. Thus, the current study was designed to capture SSI out to 60 months, with the 36-month outcomes reported currently. Device-related complications and reoperations were also recorded.

### 2.6 Analysis population

GraphPad Prism 6.01 statistical software was utilized to generate frequency counts and percentages (categorical variables) and mean ± standard deviation (continuous variables). The original Statistical Analysis Plan indicated that primary endpoints would be expressed relative to the modified Intent to Treat (mITT) population and Kaplan-Meier analysis. The Intent-to-treat (ITT) population consists of all enrolled subjects who signed the Informed Consent Form. The modified ITT (mITT) population consists of subjects in the ITT population in whom Phasix™ Mesh was implanted. Due to the extension of the original study beyond the originally planned 2 years, a higher degree of patient loss to follow up occurred than initially planned. Thus, the primary endpoints are expressed using Kaplan-Meier estimation. Other outcome measures utilized the mITT. This work complies with the (STROCC) criteria [22].

## 3 Results

### 3.1 Subject demographics

As shown in Table 1, Table 2, a total of n = 121 patients were implanted with P4HB mesh (n = 75 (62%) female) an of 54.7 ± 12.0 years old and BMI of 32.2 ± 4.5 kg/m^2^ (mean ± standard deviation). The majority were white (n = 116, 95.9%) and non-Hispanic/Latino ethnicity (n = 113, 94.3%). Comorbidities included (Table 3): obesity (78.5%), active smokers (23.1%), COPD (28.1%), diabetes mellitus (33.1%), immunosuppression (8.3%), coronary artery disease (21.5%), chronic corticosteroid use (5.0%), hypo-albuminemia (2.5%), advanced age (5.0%), and renal insufficiency (0.8%).Slightly greater than one third of the study population (34.7%) had a single comorbidity, while the remainder of the population presented with multiple comorbidities (Table 3).

**Table 1:** Flow of participants.

Flow of Patients	Patients
	(n)
**Screened**	Not Reported
**Met the initial screening criteria (ITT)**	139
**Modified intent to treat population (mITT)**	121
**Met the intraoperative inclusion/exclusion criteria**	117
**Per protocol population (PP)**	110
**Withdrew from study**	55
*Missing*	11
*Lost to Follow-Up after documented 3 attempts to contact*	21
*Subject withdrew because of an adverse event related to the study device or procedure*	4
*Subject no longer wishes to participate for non-treatment related reasons*	5
*Subject moved to an area without an active study site*	1
*Subject unable to meet follow-up requirements due to a non-study related condition*	1
*Sponsor's Decision*	1
*Death*	7
*Other*	4
**Completed study**	66

**Table 2:** Preoperative data: subject demographics and hernia diagnosis.

Subjects enrolled	n = 121
**Subjects with 36 months follow-up**	n = 82 (67.8%)
**Sex**	n = 46 (38%) male n = 75 (62%) female
**Age (years)**	54.7 ± 12.0
**Body mass index (kg/m**^2^)	32.2 ± 4.5
**Diagnosis**	Primary ventral: 14.0% Primary incisional: 44.6% Recurrent ventral: 12.4% Recurrent incisional: 28.9%

**Table 3:** Incidence of comorbid conditions in the study population.

Number of High Risk Criteria	Number of Subjects n (%)
1	42 (34.7%)
2	45 (37.2%)
3	24 (19.8%)
4	6 (5.0%)
5	3 (2.5%)
6	1 (0.8%)
Comorbid Conditions	Percentage of Subjects (%)
BMI (30–40 kg/m^2^)	78.5
Hypertension	59.5
Cardiovascular disease	34.7
Diabetes	33.1
COPD	28.1
Malignancy	24.8
Active smoker	23.1
Immunosuppression	8.3
Chronic corticosteroid use	5.0
Advanced age	5.0
Hypo-albuminemia	2.5
Renal insufficiency	0.8

### 3.2 Preoperative data

Hernia types (Table 2) included primary ventral hernia (n = 17, 14.0%), primary incisional hernia (n = 54, 44.6%), recurrent ventral hernia (n = 15, 12.4%), and recurrent incisional hernia (n = 35, 28.9%). The majority of the hernias were located at the midline (n = 102, 84.3%), and less commonly, suprapubic (n = 5, 4.1%), subxiphoid (n = 3, 2.5%), or not reported (n = 11, 9.1%).

### 3.3 Operative characteristics

Operative characteristics are shown in Table 4. Hernia defects dimensions included, length: 14.7 ± 5.6 cm, width: 8.6 ± 3.4 cm, and area:115.7 ± 80.6 cm^2^ (mean ± standard deviation) and were repaired with P4HB mesh measuring 459.38 ± 172.3 cm^2^ (mean ± standard deviation). Hernias were repaired via retrorectus technique with MR (n = 45, 37.2%), retrorectus without MR (n = 43, 35.5%), onlay without MR (n = 24, 19.8%), onlay with MR (n = 8, 6.6%), or not reported (n = 1, 0.8%). Surgical procedure time was 2.8 ± 1.4 h (mean ± standard deviation) with at least one drain placed in the majority of patients (n = 107, 88.4%).

**Table 4:** Operative data: hernia defect, procedure time, and surgical approach (MR: myofascial release).

Defect (cm^2^), mean ± SD	115.7 ± 80.6
**Mesh (cm**^**2**^**),** mean ± SD	459.38 ± 172.3
**Surgical procedure time (hrs),** mean ± SD	2.8 ± 1.4
**Surgical approach**	
Retrorectus without MR, n (%)	43 (35.5%)
Retrorectus with MR, n (%)	45 (37.2%)
Onlay without MR, n (%)	24 (19.8%)
Onlay with MR, n (%)	8 (6.6%)
Other, n (%)	1 (0.8%)

### 3.4 Postoperative outcomes

**Fig. 1:**
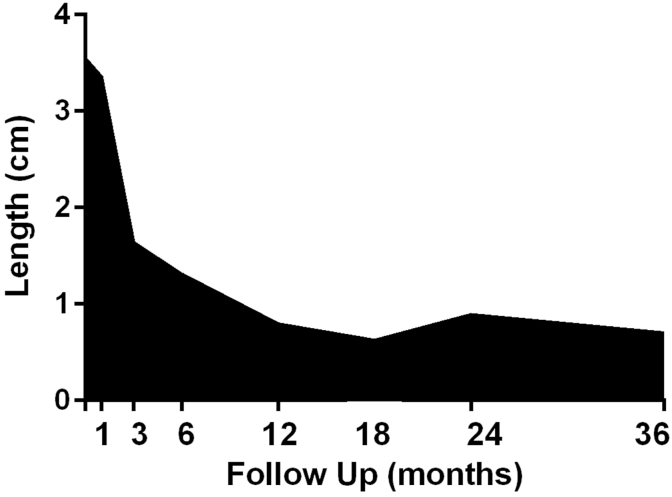
Pain Visual Analogue Scale (VAS) results depicted over time.

**Fig. 2:**
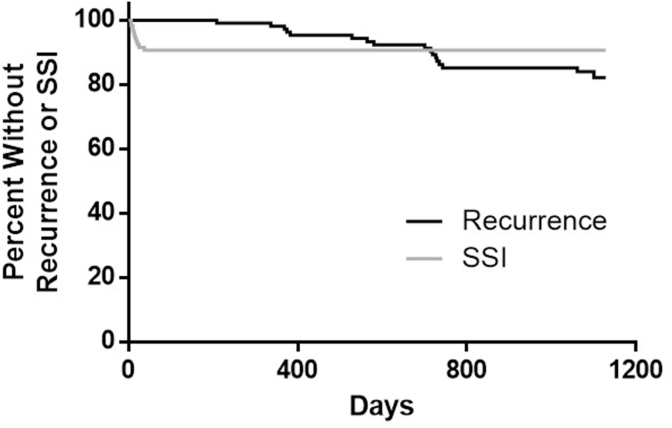
Kaplan-Meier curves for Hernia Recurrence and Surgical Site Infection (SSI).

Visual Analog Scores for pain decreased from a score of 3.55 to 0.7 (preoperatively vs. 36 months, Fig. 1). Patients averaged 5.3 ± 5.3 days (mean ± standard deviation) in the hospital (Table 5), with n = 13 (10.7%) requiring negative pressure wound therapy. Eighty-two patients (67.8%) completed 36-month follow-up, while thirty-nine patients were lost to follow-up despite phone calls, electronic communication, and mail correspondence. 17 patients experienced a hernia recurrence at 3 years (17.9% ± 0.4%), with n = 9 in the retrorectus group (12.6% ± 3.9%) and n = 8 in the onlay group (33.2% ± 10.7%). SSI (n = 11, 9.3% ± 0.03%) and seroma requiring intervention (n = 8, 6.6%) were low. The time to hernia recurrence and SSI are shown in Fig. 2. All SSIs occurred within the first six weeks postimplantation, and there were no delayed wound infections. Hernia-related complications were graded according to the Clavien-Dindo classification in Table 6 [23].

**Table 5:** Postoperative data- 3 Year Follow-Up: Primary and representative secondary outcomes.

Primary Endpoints	Hernia recurrence	17.9 ± 0.4% (n = 17)
	Surgical site infection	9.3 ± 0.03% (n = 11)

**Secondary Outcomes**	**Seroma requiring intervention**	6.6% (n = 8)
	**Rate of reoperation**	11.6% (n = 14)
	**Device-related adverse events**	15.7% (n = 19)

**Table 6:** Hernia-related complications graded according to Clavien-Dindo classification.

Clavien-Dindo Scores	Grade I	Grade II	Grade IIIa	Grade III b	Grade IVa	Grade IVb	Grade V
Hernia-Related Complications	(n)	(n)	(n)	(n)	(n)	(n)	(n)

Abdominal abscess	0	0	1	1	0	0	0
Abdominal wall disorder	1	0	0	1	0	0	0
Abdominal wound dehiscence	17	1	0	0	0	0	0
Drain complications (erythema, pain)	2	0	0	0	0	0	0
Ecchymosis	1	0	0	0	0	0	0
Epidermal necrosis	1	0	0	0	0	0	0
Erythema (incision site cellulitis, incision site erhthema, erythema)	7	0	0	0	0	0	0
Eventration (diastasis recti abdominis, hernial eventration)	4	0	0	0	0	0	0
Hematoma (incision site, intra-abdominal, post-procedural, subcutaneous)	10	1	0	0	0	0	0
Impaired healing	1	0	0	0	0	0	0
Implant site ischaemia	2	0	0	0	0	0	0
Incision site haemorrhage	3	0	0	0	0	0	0
Incision site oedema	1	0	0	0	0	0	0
Incision site vesicles	1	0	0	0	0	0	0
Infusion site urticaria	0	1	0	0	0	0	0
Necrosis (muscle, skin)	6	0	0	0	0	0	0
Pain or tenderness (abdominal, incision site, procedural)	49	2	2	0	0	0	0
Post procedural discharge	3	0	0	0	0	0	0
Postoperative wound complication	6	0	0	0	0	0	0
Postoperative wound infection	9	1	1	0	0	0	0
Seroma	15	0	0	0	0	0	0
Skin infection	1	0	0	0	0	0	0
Skin ulcer	1	0	0	0	0	0	0
Suture related complication	1	0	0	0	0	0	0
Umbilical hernia	0	0	1	1	0	0	0
Wound abscess	0	0	1	0	0	0	0
Wound complication	1	0	0	0	0	0	0
Wound dehiscence	3	0	0	0	0	0	0

## 4 Discussion

The literature has reported short- and intermediate-term outcomes data associated with hernia repair materials [16,17,24]. The current study was uniquely designed to assess outcomes along the continuum from early (1–12 months) to intermediate (18–24 months) and long-term (36–60 months) in subjects at risk for complications. The early and intermediate data associated have been published previously [8,9]. The current results provide insight into the outcomes at 36 months, with 60-month follow-up ongoing. Studies by Luijendijk and Berger have demonstrated improved outcomes for mesh-based hernia repairs relative to suture-based repairs, making mesh-based repairs the current standard of care [25,26]. While the majority of synthetic meshes result in appropriate clinical outcomes, permanent hernia mesh has been increasingly scrutinized due to long-term risk of complications such as bowel obstruction, enterocutaneous fistula, infection, seroma, hematoma, abscess, or pain [27,28]. The potential impact of a permanent synthetic mesh over the course of a lifetime has not been elucidated. An understanding of outcomes associated with absorbable mesh provides a useful framework for patient discussions.

Several clinical studies of P4HB mesh have been reported, primarily at 18-months [[7], [8], [9]]. In two studies, P4HB mesh was utilized in onlay or retrorectus position, with or without myofascial release (MR). Hernia recurrence rates and surgical site occurrences (SSO) requiring intervention, including surgical site infection (SSI), seroma, wound dehiscence, skin necrosis, hematoma, and fistula were reported. In the Plymale study, 31 subjects underwent VIHR with P4HB mesh. At a median follow-up of 414 days (~13.8 months), 0% hernia recurrence and 19% SSO (12.9% seroma, 3.2% abdominal necrosis, and 3.2% wound dehiscence) were reported [9]. In the 18-month reporting of the current trial, P4HB mesh was utilized to perform VIHR in 121 subjects [8]. Consistent with clinical trials with similar follow-up, outcomes were favorable, including: 9% hernia recurrence, 9% SSI, and 6% seroma. In the Levy study, P4HB mesh was utilized in a highly morbid study population of n = 105 patients undergoing component separation with onlay P4HB [7]. Eighteen patients (n = 18, 17%) experienced a hernia recurrence at a mean of 18 months follow-up (range: 2–36 months). Low rates of infection (n = 5, 5%) and seroma (n = 6, 6%) were also reported. To date, long-term clinical outcomes associated with P4HB mesh have not been reported beyond these 18–24 month studies, possibly due to the high costs associated with conducting a long-term clinical trial. Thus, the current study extends the previously published study and reports the 3-year postoperative outcomes associated with P4HB mesh in the same prospective, multicenter trial in subjects at risk of postoperative complications. This study provides important insight into the long-term performance of P4HB mesh at a time point in which the mesh itself is no longer contributing to the mechanical strength of the repair. At 36 months postimplantation, all of the repair strength is dependent upon the strength of the native abdominal wall in combination with the host tissue that has been regenerated at the repair site.

The results of the current study revealed 17.9% ± 0.4% hernia recurrence, 6.6% seroma, and 9.3% ± 0.03% SSI at 36 months. Importantly, all SSIs occurred within the first six weeks postimplantation, and there were no delayed wound infections. These results compare well with several other published studies. In a prospective, randomized study comparing mesh repair to suture repair without mesh, Luijendijk et al. reported 24% recurrence at 3 years for mesh repairs compared to 43% recurrence for suture repairs [26]. In a prospective, Danish, nationwide hernia database study, Helgstrand et al. reported outcomes after elective incisional hernia repair via onlay, retrorectus, or intraperitoneal mesh placement [29]. With a long-term median follow-up of 48 months, Helgstrand et al. observed an 18.3% overall hernia recurrence rate and 9.5% reoperation rate comparable to the current study (17.9% and 11.6%, respectively). A 21% recurrence rate was reported for open repairs, which is greater than the current study. Onlay repairs had a hazard ratio of 1.7, consistent with the current study. In a prospective, randomized controlled trial, Sevinc et al. evaluated outcomes associated with onlay versus retrorectus mesh placement for incisional hernia repair, with 50 subjects in each group [30]. At a median follow-up of 37.1 months, Sevinc et al. observed a 4% incidence of SSI and an 8% incidence of seroma, which compare well to the current study at 9.3% ± 0.03% and 6.6%, respectively. However, the subjects in the Sevinc et al. study had a BMI of 25.9 ± 3.5 kg/m^2^ and hernia defect of 73.4 ± 66.3 cm^2^, while patients in the current study had a BMI of 32.2 ± 4.5 kg/m^2^ and hernia defect of 115.7 ± 80.6 cm^2^.

When the results of the current study were separated into onlay versus retrorectus repairs, the recurrence rates were 2.5 times higher in the onlay group compared to the retrorectus group, with an overall recurrence rate of 17.9% ± 0.4%. Several studies have demonstrated similar ratios of 2–3 times greater onlay recurrence rates compared to retrorectus recurrence rates, comparable to the current study [[30], [31], [32], [33], [34], [35]]. In a systematic review of the literature, Sosin et al. compared outcomes and complications associated with onlay, retrorectus, interposition, and underlay ventral hernia repair techniques. At a mean follow-up of 37.5 months, they reported an overall mean recurrence rate of 8.3% (12.9% onlay and 5.8% retrorectus, ratio of 2.2). SSI and seroma/hematoma were 11.1% and 11.3%, respectively, which compare well with the current data. In another systematic review and meta-analysis, Holihan et al. evaluated hernia recurrence and SSI associated with open ventral hernia repair [33]. With follow-up ranging from 12 to 98 months, Holihan et al. reported a significantly higher recurrence rate (16.5% vs. 7%, OR 0.218) and incidence of SSI (16.9% vs. 3.7%, OR 0.449) for onlay repairs compared to retrorectus repairs (2.4 times greater recurrences for onlay vs. retrorectus). Finally, Levy et al. reported a recurrence rate of 17% for onlay P4HB mesh repair with component separation in a highly morbid study population in which 91% had one or more major comorbidities [7].

This study represents a prospective, multicenter trial with long-term follow-up rather than a randomized controlled trial. Several other peer-reviewed studies have evaluated other resorbable synthetic or biologic hernia repair materials such as TIGR® Matrix [15], Gore® Bio-A® [36], and Strattice™ [17] in similar single arm studies, albeit with shorter follow-up periods. Historically, randomized controlled trials in hernia repair have primarily focused on technique rather than biomaterial comparisons [25,37,38]. However, head-to-head comparisons of devices are commonly addressed in animal studies. Several important limitations of the current study should be acknowledged, including the lack a control group and the fact that all of the patients had Class I (clean) wounds. As such, the data cannot be directly compared to other biomaterials or other wound types. However, the data provide important insight into the long-term performance of P4HB mesh at a time point in which the mesh itself is no longer contributing to the mechanical strength of the repair. At 36 months postimplantation, all of the repair strength is dependent upon the strength of the native abdominal wall in combination with the host tissue that has been regenerated at the repair site. Future prospective randomized trials comparing outcomes to other biomaterials are needed to understand outcomes relative to other biomaterials.

## 5 Conclusion

Long-term outcomes following VIHR with P4HB mesh demonstrate low recurrence rates at the 3-years (36-months). 5-year (60-month) follow-up is ongoing.

## Funding

This study was funded by CR Bard/Becton Dickinsone. Authors were reimbursed for expenses related to the conduct of the study.

## Provenance and peer review

Not commissioned, externally peer-reviewed.

## Ethical approval

This study was registered at ClinicalTrials.gov (NCT01961687). The protocol was approved by the Institutional Review Board (IRB) at each institution prior to enrolling subjects. All subjects provided informed consent prior to enrollment in the study.

## Consent

All subjects provided informed consent prior to enrollment in the study.

## Author contribution

**Study Conception/Design:** Roth.

**Data Collection:** Roth, Anthone, Selzer, Poulose, Pierce, Bittner, Hope, Dunn, Martindale, Goldblatt, Earle, Romanelli, Mancini, Greenberg, Linn, Parra-Davila, Sandler, Salluzzo, Voeller.

**Data Analysis/Interpretation:** Roth, Anthone, Selzer, Poulose, Pierce, Bittner, Hope, Dunn, Martindale, Goldblatt, Earle, Romanelli, Mancini, Greenberg, Linn, Parra-Davila, Sandler, Deeken, Verbarg, Salluzzo, Voeller.

**Drafting of Manuscript:** Roth, Anthone, Selzer, Poulose, Pierce, Bittner, Hope, Dunn, Martindale, Goldblatt, Earle, Romanelli, Mancini, Greenberg, Linn, Parra-Davila, Sandler, Deeken, Verbarg, Salluzzo, Voeller.

**Final Approval of Manuscript:** Roth, Anthone, Selzer, Poulose, Pierce, Bittner, Hope, Dunn, Martindale, Goldblatt, Earle, Romanelli, Mancini, Greenberg, Linn, Parra-Davila, Sandler, Deeken, Verbarg, Salluzzo, Voeller.

## Registration of research studies

1. Name of the registry: Clinicaltrials.gov.

2. Unique Identifying number or registration ID: NCT01961687.

3. Hyperlink to your specific registration (must be publicly accessible and will be checked): https://clinicaltrials.gov/ct2/show/NCT01961687?term=NCT01961687&draw=2&rank=1.

## Guarantor

Dr. Roth.

## Declaration of competing interest

Dr. Roth reports a grant and consulting fees from C.R. Bard, Inc./Davol/Becton Dickinson (BD) during the conduct of this study. Dr. Roth also reports consulting fees (Johnson & Johnson) and stock (Miromatrix) outside of the current work.

Dr. Anthone has no conflicts of interest to disclose related to this study. Dr. Anthone reports consulting fees and speaker honoraria (Becton Dickinson (BD)) outside of the current work.

Dr. Selzer reports a grant from C.R. Bard, Inc./Davol/Becton Dickinson (BD) during the conduct of the study. Dr. Selzer also reports consulting fees (Cook Biotech, Inc.) outside of the current work.

Dr. Poulose has no conflicts of interest to disclose related to the current study. Dr. Poulose reports salary support (Americas Hernia Society Quality Collaborative) and research support (Becton Dickinson (BD) and Advanced Medical Solutions) outside of the current work.

Dr. Pierce has no conflicts of interest to disclose related to the current study. Dr. Pierce reports research support (Intuitive Surgical Solutions) outside of the current work and his spouse is a salaried employee of a medical device company (CareFusion) that falls under the overall umbrella of the parent company, Becton Dickinson (BD).

Drs. Dunn, Bittner, Linn, Romanelli, and Sandler report a grant from C.R. Bard, Inc./Davol/Becton Dickinson (BD) during the conduct of the study but have nothing to disclose outside of the current work.

Dr. Hope reports consulting fees and research support from C.R. Bard, Inc./Davol/Becton Dickinson (BD) during the conduct of the study. Dr. Hope also reports consulting fees, honoraria, and research support (Intuitive, W.L. Gore, and Medtronic) outside of the current work.

Dr. Martindale has no conflicts of interest to disclose related to the current study. Dr. Martindale reports consulting fees (Allergan, C.R. Bard, Inc./Davol/Becton Dickinson (BD), and Nestle) and employment (OHSU) outside of the current work.

Dr. Goldblatt reports a grant from C.R. Bard, Inc./Davol/Becton Dickinson (BD) during the conduct of the study. Dr. Goldblatt also reports consulting fees (W.L. Gore, Medtronic, and Allergan) and a grant (Medtronic) outside of the current work.

Dr. Earle has no conflicts of interest to disclose related to the current study. Dr. Earle reports consulting fees (Becton Dickinson (BD) and Medtronic) and ownership stake/advisory board (Via Surgical) outside of the current work.

Dr. Mancini has no conflicts of interest to disclose related to the current study. Dr. Mancini reports consulting fees (Stryker Endoscopy) and speakers’ fees (Gore Medical and Medtronic) outside of the current work.

Dr. Greenberg reports a grant from C.R. Bard, Inc./Davol/Becton Dickinson (BD) during the conduct of the study. Dr. Greenberg also reports grants (Becton Dickinson (BD) and Medtronic), as well as course registration, travel, and lodging (Intuitive) outside of the current work.

Dr. Parra-Davila has no conflicts of interest to disclose related to the current study. Dr. Parra-Davila reports consulting fees (C.R. Bard, Inc./Davol/Becton Dickinson (BD), Intuitive, Auris, Johnson & Johnson, and Titan), board membership (C.R. Bard, Inc./Davol/Becton Dickinson (BD)), and proctoring (Intuitive) outside of the current work.

Dr. Deeken reports consulting fees from C.R. Bard, Inc./Davol/Becton Dickinson (BD) during the conduct of the study. Dr. Deeken also reports consulting fees from C.R. Bard, Inc./Davol/Becton Dickinson (BD), Surgical Innovation Associates, Americas Hernia Society Quality Collaborative, Colorado Therapeutics, TelaBio, and Aran Biomedical outside the submitted work, as well as grants from Ethicon, Inc. and TelaBio to support a book chapter outside the submitted work. In addition, Dr. Deeken is the owner of Covalent Bio, LLC and holds the following issued patents: 2009293001, 2334257, 2,334,257UK, 602009046407.8, 2,334,257FR, 16/043,849 and 2,737,542.

Dr. Verbarg reports consulting fees from C.R. Bard, Inc./Davol/Becton Dickinson (BD) during the conduct of the study. Dr. Verbarg has no other conflict of interest to disclose outside of the current work.

Drs. Salluzzo and Voeller have no conflicts of interest to disclose relative to the current study or any other work.

## References

[1] Deeken C.R., Lake S.P. (2017). Mechanical properties of the abdominal wall and biomaterials utilized for hernia repair. J. Mech. Behav. Biomed. Mater..

[2] Miserez M., Jairam A.P., Boersema G.S.A., Bayon Y., Jeekel J., Lange J.F. (2019). Resorbable synthetic meshes for abdominal wall defects in preclinical setting: a literature review. J. Surg. Res..

[3] Karp N., Choi M., Kulber D.A., Downey S., Duda G., Kind G. (2017). SERI Surgical Scaffold in 2-stage breast reconstruction: 2-year data from a prospective multicenter trial. Plast. Reconstr. Surg. Glob. Open.

[4] Rice R.D., Ayubi F.S., Shaub Z.J., Parker D.M., Armstrong P.J., Tsai J.W. (2010). Comparison of Surgisis, AlloDerm, and Vicryl Woven Mesh grafts for abdominal wall defect repair in an animal model. Aesthetic Plast. Surg..

[5] Williams S.F., Rizk S., Martin D.P. (2013). Poly-4-hydroxybutyrate (P4HB): a new generation of resorbable medical devices for tissue repair and regeneration. Biomed. Tech..

[6] Messa C.A., Kozak G., Broach R.B., Jp F. (2019). When the mesh goes away: an analysis of poly-4-hydroxybutyrate mesh for complex hernia repair. Plast. Reconstr. Surg. Glob. Open.

[7] Levy A.S., Bernstein J.L., Premaratne I.D., Rohde C.H., Otterburn D.M., Morrison K.A. (2020). Poly-4-hydroxybutyrate (Phasix) mesh onlay in complex abdominal wall repair.

[8] Roth J.S.A.G., Selzer D.J., Poulose B.K., Bittner J.G., Hope W.W., Dunn R.M. (2018). Prospective evaluation of poly-4-hydroxybutyrate mesh in CDC Class I/high risk ventral and incisional hernia repair: 18 Months follow-up. Surg. Endosc..

[9] Plymale M.A., Davenport D.L., Dugan A., Zachem A., Roth J.S. (2017). Ventral hernia repair with poly-4-hydroxybutyrate mesh.

[10] Tollens Jørgensen, Vries d, Köckerling, Piessen, Windsor (September. 2019). A post-market, prospective, multi-center, single-arm clinical investigation of Phasix Mesh for VHWG Grade 3 midline incisional henria repair: 18 month outcomes in patients in the European Union. Accepted as an Oral Presentation at the European Hernia Society Congress in Hamburg.

[11] Deeken C.R., Matthews B.D. (2013). Characterization of the mechanical strength, resorption properties, and histologic characteristics of a fully absorbable material (poly-4-hydroxybutyrate-PHASIX Mesh) in a porcine model of hernia repair. ISRN Surg..

[12] Martin D.P., Badhwar A., Shah D.V., Rizk S., Eldridge S.N., Gagne D.H. (2013). Characterization of poly-4-hydroxybutyrate mesh for hernia repair applications. J. Surg. Res..

[13] Rhemtulla I.A., Mauch J.T., Broach R.B., Messa C.A., Fischer J.P. (2018 Sep). Prophylactic mesh augmentation: patient selection, techniques, and early outcomes. Am. J. Surg..

[14] Scott J.R., Deeken C.R., Martindale R.G., Rosen M.J. (2016). Evaluation of a fully absorbable poly-4-hydroxybutyrate/absorbable barrier composite mesh in a porcine model of ventral hernia repair. Surg. Endosc..

[15] Ruiz-Jasbon F., Norrby J., Ivarsson M.L., Bjorck S. (2014). Inguinal hernia repair using a synthetic long-term resorbable mesh: results from a 3-year prospective safety and performance study. Hernia.

[16] Rosen M., Bauer J., Harmaty M., Carbonell A.M., Cobb W.S., Matthews B.D. (2017). Multicenter, prospective, longitudinal study of the recurrence, surgical site infection, and quality of life after contaminated ventral hernia repair using biosynthetic absorbable mesh: the COBRA Study. Ann. Surg..

[17] Itani K.M., Rosen M., Vargo D., Awad S.S., Denoto G., Butler C.E. (2012). Prospective study of single-stage repair of contaminated hernias using a biologic porcine tissue matrix: the RICH Study. Surgery.

[18] CDC surgical site infection event protocol. https://wwwcdcgov/nhsn/pdfs/pscmanual/9pscssicurrentpdf.

[19] Mangram A., Horan T., Pearson M., Silver L., Jarvis W. (1999). Guideline for prevention of surgical site infection. Am. J. Infect. Contr..

[20] Whitehead-Clarke T., Windsor A. (2020). Surgical Site Infection: the Scourge of Abdominal Wall Reconstruction.

[21] Delikoukos S., Tzovaras G., Liakou P., Mantzos F., Hatzitheofilou C. (2007). Late-onset deep mesh infection after inguinal hernia repair. Hernia.

[22] Agha R.A.-R.A., Crossley E., Dowlut N., Iosifidis C., Mathew G., for the STROCSS Group (2019). The STROCSS 2019 guideline: strengthening the reporting of cohort studies in surgery. Int. J. Surg..

[23] Dindo D.D.N., Clavien P.-A. (2004). Classification of surgical complications. A new proposal with evaluation in a cohort of 6336 patients and results of a survey. Ann. Surg..

[24] Carbonell A.M., Criss C.N., Cobb W.S., Novitsky Y.W., Rosen M.J. (2013). Outcomes of synthetic mesh in contaminated ventral hernia repairs. J. Am. Coll. Surg..

[25] Burger J.W., Luijendijk R.W., Hop W.C., Halm J.A., Verdaasdonk E.G., Jeekel J. (2004). Long-term follow-up of a randomized controlled trial of suture versus mesh repair of incisional hernia. Ann. Surg..

[26] Luijendijk R.W., Hop W.C., van den Tol M.P., de L D.C., Braaksma M.M., IJzermans J.N. (2000). A comparison of suture repair with mesh repair for incisional hernia. N. Engl. J. Med..

[27] Leber G.E., Garb J.L., Alexander A.I., Reed W.P. (1998). Long-term complications associated with prosthetic repair of incisional hernias. Arch. Surg..

[28] Kokotovic D., Bisgaard T., Helgstrand F. (2016). Long-term recurrence and complications associated with elective incisional hernia repair. J. Am. Med. Assoc..

[29] Helgstrand F., Rosenberg J., Kehlet H., Jorgensen L.N., Bisgaard T. (2013). Nationwide prospective study of outcomes after elective incisional hernia repair. J. Am. Coll. Surg..

[30] Sevinc B., Okus A., Ay S., Aksoy N., Karahan O. (2018). Randomized prospective comparison of long-term results of onlay and sublay mesh repair techniques for incisional hernia. Turk. J. Surg..

[31] Sosin M., Nahabedian M.Y., Bhanot P. (2018). The perfect plane: a systematic review of mesh location and outcomes, update 2018. Plast. Reconstr. Surg..

[32] Demetrashvili Z., Pipia I., Loladze D., Metreveli T., Ekaladze E., Kenchadze G. (2017). Open retromuscular mesh repair versus onlay technique of incisional hernia: a randomized controlled trial. Int. J. Surg..

[33] Holihan J.L., Nguyen D.H., Nguyen M.T., Mo J., Kao L.S., Liang M.K. (2016). Mesh location in open ventral hernia repair: a systematic review and network meta-analysis. World J. Surg..

[34] Israelsson L.A., Smedberg S., Montgomery A., Nordin P., Spangen L. (2006). Incisional hernia repair in Sweden 2002. Hernia.

[35] de Vries Reilingh T.S., van Geldere D., Langenhorst B., de Jong D., van der Wilt G.J., van Goor H. (2004). Repair of large midline incisional hernias with polypropylene mesh: comparison of three operative techniques. Hernia.

[36] Rosen M.J., Bauer J.J., Harmaty M., Carbonell A.M., Cobb W.S., Matthews B. (2017). Multicenter, prospective, longitudinal study of the recurrence, surgical site infection, and quality of life after contaminated ventral hernia repair using biosynthetic absorbable mesh: the COBRA study. Ann. Surg..

[37] Jairam A.P., Timmermans L., Eker H.H., Pierik R., van Klaveren D., Steyerberg E.W. (2017). Prevention of incisional hernia with prophylactic onlay and sublay mesh reinforcement versus primary suture only in midline laparotomies (PRIMA): 2-year follow-up of a multicentre, double-blind, randomised controlled trial. Lancet.

[38] Itani K.M., Hur K., Kim L.T., Anthony T., Berger D.H., Reda D. (2010). Comparison of laparoscopic and open repair with mesh for the treatment of ventral incisional hernia: a randomized trial. Arch. Surg..

